# The Detection of Potential Native Probiotics *Lactobacillus* spp. against *Salmonella* Enteritidis, *Salmonella* Infantis and *Salmonella* Kentucky ST198 of Lebanese Chicken Origin

**DOI:** 10.3390/antibiotics11091147

**Published:** 2022-08-24

**Authors:** Rima El Hage, Jeanne El Hage, Selma P. Snini, Imad Ammoun, Joseph Touma, Rami Rachid, Florence Mathieu, Jean-Marc Sabatier, Ziad Abi Khattar, Youssef El Rayess

**Affiliations:** 1 Food Microbiology Laboratory, Lebanese Agricultural Research Institute (LARI), Fanar Station, Jdeideh El-Metn P.O. Box 901965, Lebanon; 2 Laboratoire de Génie Chimique, UMR 5503 CNRS/INPT/UPS, INP-ENSAT, 1, Université de Toulouse, Avenue de l’Agrobiopôle, 31326 Castanet-Tolosan, France; 3 Animal Health Laboratory, Lebanese Agricultural Research Institute (LARI), Fanar Station, Jdeideh El-Metn P.O. Box 901965, Lebanon; 4 Milk and Milk Products Laboratory, Lebanese Agricultural Research Institute (LARI), Fanar Station, Jdeideh El-Metn P.O. Box 901965, Lebanon; 5 CNRS UMR 7051, INP, Inst Neurophysiopathol, Aix-Marseille Université, 13385 Marseille, France; 6 Microbiology/Tox-Ecotoxicology Team, Laboratory of Georesources, Geosciences and Environment (L2GE), Faculty of Sciences 2, Lebanese University, Campus Fanar, Jdeideh El-Metn P.O. Box 90656, Lebanon; 7 Faculty of Agricultural and Food Sciences, Holy Spirit University of Kaslik, Jounieh P.O. Box 446, Lebanon

**Keywords:** *Salmonella* spp., poultry, probiotic, *Ligilactobacillus salivarius*, inhibition, adhesion

## Abstract

*Salmonella* continues to be a major threat to public health, especially with respect to strains from a poultry origin. In recent years, an increasing trend of antimicrobial resistance (AMR) in *Salmonella* spp. was observed due to the misuse of antibiotics. Among the approaches advised for overcoming AMR, probiotics from the *Lactobacillus* genus have increasingly been considered for use as effective prophylactic and therapeutic agents belonging to the indigenous microbiota. In this study, we isolated lactobacilli from the ilea and ceca of hens and broilers in order to evaluate their potential probiotic properties. Four species were identified as *Limosilactobacillus* *reuteri* (*n* = 22, 45.8%), *Ligilactobacillus* *salivarius* (*n* = 20, 41.6%), *Limosilactobacillus fermentum* (*n* = 2, 4.2%) and *Lactobacillus crispatus* (*n* = 1, 2%), while three other isolates (*n* = 3, 6.25%) were non-typable. Eight isolates, including *Ligilactobacillus* *salivarius* (*n* = 4), *Limosilactobacillus* *reuteri* (*n* = 2), *L. crispatus* (*n* = 1) and *Lactobacillus* spp. (*n* = 1) were chosen on the basis of their cell surface hydrophobicity and auto/co-aggregation ability for further adhesion assays using the adenocarcinoma cell line Caco-2. The adhesion rate of these strains varied from 0.53 to 10.78%. *Ligilactobacillus* *salivarius* A30/i26 and 16/c6 and *Limosilactobacillus reuteri* 1/c24 showed the highest adhesion capacity, and were assessed for their ability to compete in and exclude the adhesion of *Salmonella* to the Caco-2 cells. Interestingly, *Ligilactobacillus* *salivarius* 16/c6 was shown to significantly exclude the adhesion of the three *Salmonella* serotypes, *S*. Enteritidis, *S*. Infantis and *S*. Kentucky ST 198, to Caco-2 cells. The results of the liquid co-culture assays revealed a complete inhibition of the growth of *Salmonella* after 24 h. Consequently, the indigenous *Ligilactobacillus* *salivarius* 16/c6 strain shows promising potential for use as a preventive probiotic added directly to the diet for the control of the colonization of *Salmonella* spp. in poultry.

## 1. Introduction

Non-typhoidal *Salmonella* strains are the leading cause of foodborne gastroenteritis [1]. Poultry products are primarily consumed worldwide and are commonly known to be reservoirs for a variety of microorganisms. *Salmonella* is the most frequently encountered pathogen in poultry products, as well as the most prominent inhabitant of avian gastrointestinal tracts (GIT) [2]. In developing countries, a high prevalence of *Salmonella* has been recorded, ranging from 13% to 39% in South America, estimated at 35% in Africa, and ranging from 35% to 50% in Asia [3]. In Lebanon, according to our recent study, the percentage of contamination of poultry meat at the retail level (supermarkets and restaurants) was estimated at 22.4% [4].

Several control strategies have been adopted to reduce or eliminate *Salmonella* at the farm level. It is well known that the use of antibiotic growth promoters (AGPs) and other prophylactic treatments improve animal health and productivity rates in livestock farming [5]. However, the mass use of antibiotics as feed additives has led to the emergence and spread of antimicrobial resistant pathogens and epidemic multi-drug-resistant clones and/or resistance genes in poultry reservoirs [6]. Recently, resistance to critical antibiotics, namely fluoroquinolones and expanded-spectrum β-lactam antibiotics, has spread worldwide and reached humans through the food chain [7]. Consequently, since 2006, AGP use in the animal industry has been completely banned in the EU [8] and reduced in many other countries [9]. However, in Lebanon, there are no current regulations concerning the use of AGPs in animal husbandry (personal communication with the ministry of agriculture).

Many countries have also implemented control programs to tackle *Salmonella* in poultry farms. Such was the case in the USA (National Poultry Improvement Plan (NPIP) for the eradication of *Salmonella* in eggs (1989) and meats (1994)) and the EU (Commission Regulation (EC), No. 2160/2003). These measurements ultimately led to the successful reduction of targeted *Salmonella* spp., but unfortunately cleared the way for the emergence of more resistant, less common serotypes, such as *S*. Heidelberg and *S*. Kentucky [10].

A promising alternative strategy against enteropathogens is the use of lactic acid bacteria (LAB) as probiotics. Probiotics are non-pathogenic live microorganisms which confer health benefits on their host when ingested in adequate quantities [11]. The use of (direct-fed microbial) probiotics as broiler growth promoters [12,13] could improve livestock health and might reduce the emergence of antimicrobial resistance (AMR) [14]. Strains of *Lactobacillus* spp. and *Bifidobacterium* spp. are the most widely studied probiotics acting against gastrointestinal microbial pathogens [15], especially against *Salmonella* infections in the broiler gastrointestinal tract [16,17]. Two fundamental mechanisms of inhibition of pathogenic microorganisms have been described, namely the direct cell competitive exclusion and the production of inhibitory compounds, including lactic and acetic acids, hydrogen peroxide, bacteriocin or bacteriocin-like inhibitors, and fatty and amino acid metabolites [18].

Intestinal adhesion and colonization are the first steps of the *Salmonella* infection process in poultry. Therefore, the adhesion ability is an essential prerequisite of, and one of the main criteria for selecting, potential probiotic strains [11]. The probiotic ability prevents the selected strains from undergoing direct elimination by peristalsis and inhibits the colonization of enteric pathogens in chickens by competitive exclusion [19]. Methods of evaluating the capacity of LAB to adhere to poultry epithelia may include in vitro analysis of, for example, cell aggregation, cell wall hydrophobicity, and adhesion to the human colorectal adenocarcinoma cell line (Caco-2) and chicken hepatocellular carcinoma cell line (LMH) [12]. Since bacterial populations of GIT are specific to their animal hosts, poultry-derived probiotics could be more effective than non-specific microbial agents [20].

This study aims to develop an effective probiotic derived from broiler and chicken GITs. In this regard, in vitro experiments were conducted to reveal the probiotic activity of native poultry-derived lactobacilli strains against the most relevant and drug-resistant *Salmonella* spp. (*S*. Enteritidis, *S*. Infantis and *S*. Kentucky ST198) in Lebanese poultry farms. The screening of lactobacilli strains for their anti-*Salmonella* activity, safety and surface probiotic properties was also carried out. Finally, the lactobacilli showing a great probiotic potential were selected for the further in vitro characterization of their adhesion ability and kinetics in co-culture. In fact, their adhesion and abilities to exclude and compete with *Salmonella* serotypes in epithelial tissues, using the Caco-2 cell line as an experimental model, were evaluated, as well as their capacity to inhibit pathogen growth in a mixed co-culture model.

## 2. Results

### 2.1. Screening of Lactobacilli and Their Anti-Salmonella Activity

In total, 210 stains (155 from the 16 trials and 55 from commercial birds) which presented as gram-positive bacilli/coccobacilli with no catalase activity were collected from broiler ceca and ileum samples. All lactobacilli were found to produce inhibition zones against the three serotypes of *Salmonella* based on the agar spot-on-lawn assay. The radii of their inhibition zones ranged from 1.2 to 4.4 cm (data not shown). However, the cell-free supernatants (CFSs) of all lactobacilli, neutralized to pH 6.8, did not display any antimicrobial effects against the *Salmonella* serotypes studied.

### 2.2. Genotypic Identification of Lactobacilli Isolates with Phylogenetic Relations

**Figure 1:**
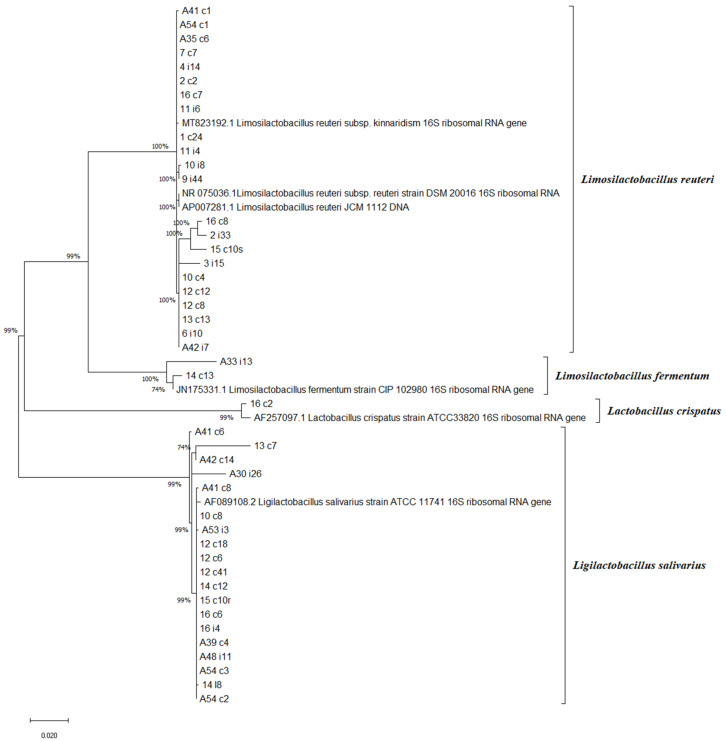
A maximum-likelihood phylogenetic tree reconstructed using 16S rRNA gene sequences. The percentage of replicate trees in which the associated species clustered together in the bootstrap test (1000 replicates) are shown next to the branches [21]. Evolutionary analyses were conducted in MEGA X. *Limosilactobacillus fermentum* comb. nov. CIP 102980 (JN175331), *Lactobacillus crispatus* ATCC 33820T (AF257097), *Ligilactobacillus salivarius* comb. nov. ATCC 11741T (AF089108), *Limosilactobacillus reuteri* comb. nov. JCM 1112 (AP007281), *Limosilactobacillus reuteri subsp.kinnaridis* (MT823192), and *Limosilactobacillus reuteri subsp. reuteri* DSM20016 (NR075036) were selected as type strains. The 16S rRNA gene accession numbers are provided in parentheses.

Lactobacilli strains (*n* = 48) were chosen according to their high anti-*Salmonella* activity in the spot-on-lawn test. The 16S rRNA gene sequence analysis identified four species: *Limosilactobacillus reuteri* (formerly *Lactobacillus reuteri*) (*n* = 22, 45.83%), *Ligilactobacillus salivarius* (formerly *Lactobacillus salivarius*) (*n* = 20, 41.66%), *Limosilactobacillus fermentum* (formerly *Lactobacillus fermentum*) (*n* = 2, 4.16%) and *Lactobacillus crispatus* (*n* = 1, 2%) (Figure 1). The three remaining isolates (16/i10, 14/i15, A30/c2, 6.25%) were non-typable. The most common species were *Limosilactobacillus reuteri* and *Ligilactobacillus* *salivarius*. The phylogenetic tree demonstrated a close relation among the same species. However, we could not obtain a better strain resolution at the subspecies level among *Limosilactobacillus reuteri*. To gain further insight into the genetic dissimilarities and evolutionary relationships among the lineages isolated here would require profound core-gene-based phylogenetic analyses. These analyses are not considered here, since the focus of our study is the probiotic potential of lactobacilli strains.

### 2.3. Analysis of Surface Properties

**Figure 2:**
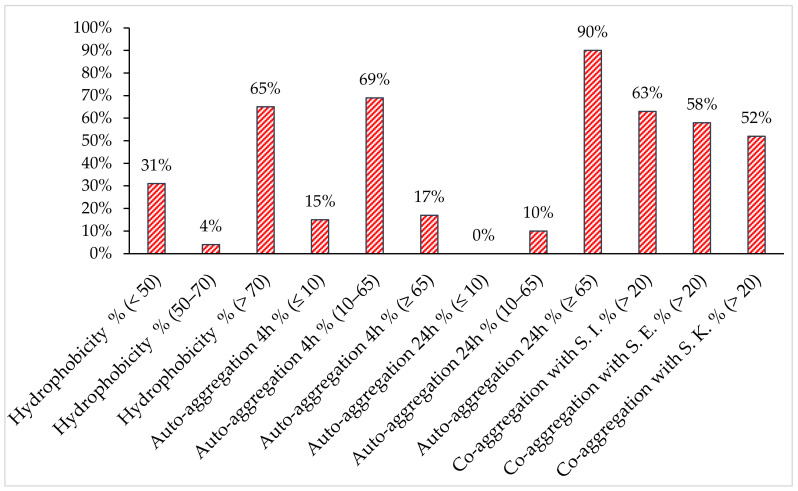
Isolate distribution in defined ranges of percentage of hydrophobicity, auto-aggregation and co-aggregation with the three *Salmonella* spp. (*S*. Enteritidis (S.E.), *S*. Kentucky ST198 (S.K.) and *S*. Infantis (S.I.).

The visual screening of the forty-eight chosen lactobacilli isolates showed that most of the strains were Agg+/Agg− (75%), while Agg+ and Agg− represented 14,6% and 10.4%, respectively (data not shown). These results were confirmed by auto-aggregation assays at 4 h. As shown in Figure 2, category I demonstrated a significant auto-aggregation percentage (≥65%) compared to category II (≤10%), while category III ranged from 10 to 65% except for three strains: one > 65% and two ≤ 10%. Auto-aggregation was determined in all the lactobacilli tested (*n* = 45, 90%) at 24 h.

The co-aggregation properties of the lactobacilli strains with *Salmonella* serotypes differed among the strains and ranged from 0 to 94.6% (data not shown). They co-aggregated with *S*. Kentucky ST198, *S*. Enteritidis and *S*. Infantis at 52%, 58% and 63%, respectively. Otherwise, a high affinity for xylene was shown (65%) compared to non-hydrophobic isolates (31%).

### 2.4. Hydrophobicity and Auto/co-Aggregation Correlation

**Figure 3:**
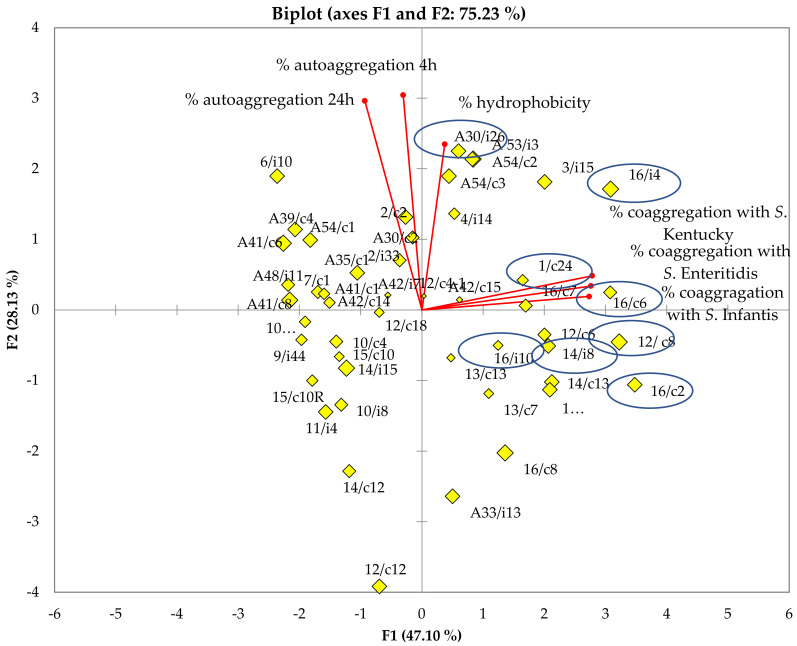
Graphic representation of the principal component analysis (PCA) of surface proprieties, including hydrophobicity and auto/co-aggregation, for the 48 lactobacilli isolated stains. The selected stains are encircled.

The results obtained from the analysis of the lactobacilli surfaces were subjected to principal component analysis (PCA) (Figure 3). The first PC1 and second PC2 principal components could explain 47.1% and 28.13% of the total variance, respectively. Based on the cell surface properties, eight lactobacilli strains were chosen for further adhesion assays, whose characteristics are summarized in Table 1. *Ligilactobacillus* *salivarius* A30/i26 was shown to be highly hydrophobic (98.84% ± 1.34), possessing an aggregation phenotype (Agg+) and an ability to aggregate rapidly at 4 h (76.15% ± 3.93). The most co-aggregative strains were *L. crispatus* 16/c2, *Limosilactobacillus reuteri* 12/c8 and *Ligilactobacillus* *salivarius* (16/c4, 16/c6 and 14/i8). In addition to these properties, *Ligilactobacillus* *salivarius* 16/c6 did not exhibit auto-aggregation at 4 h but only at 24 h (9.89% ± 3.63 and 95.91% ± 2.58, respectively). However, *Ligilactobacillus* *salivarius* 16/c4 displayed an aggregation phenotype (Agg+) and rapidly auto-aggregated at 4 h (76.23% ± 3.38). Both *Lactobacillus* spp. 16/i10 and *Limosilactobacillus reuteri* 1/c24 displayed high hydrophobicity levels (98.36% ± 3.63 and 91.81% ± 7.78, respectively); however, they either did not show an auto-aggregation capacity at 4 h, or only did so to a moderate degree (6.16% ± 5.53 and 13.76% ± 1.87, respectively) (Table 1).

There was no significant correlation between hydrophobicity, auto-aggregation, and co-aggregation among the forty-eight strains tested (Table 2). On the contrary, a significant correlation was detected (*p* < 0.05) between the co-aggregation results of the three *Salmonella* serotypes with lactobacilli isolates, since the correlation coefficient value reached up to 0.890.

### 2.5. Assays for Tolerance to Simulated Gastrointestinal Conditions of Chickens

**Figure 4:**
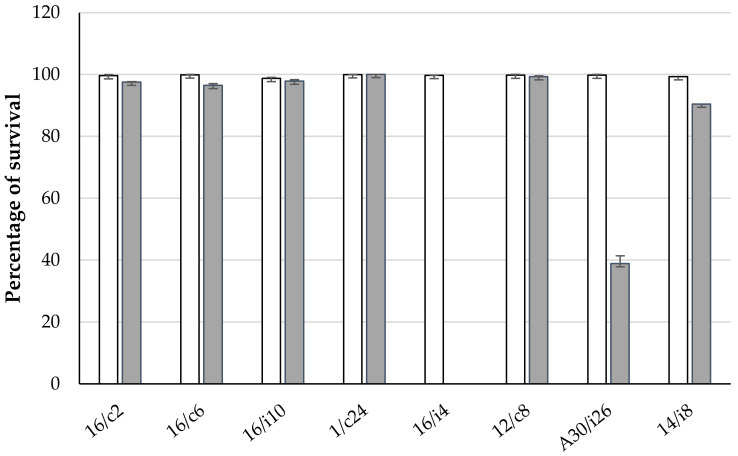
Effects of simulated gastrointestinal conditions on lactobacilli viability. White and grey columns correspond to lactobacilli subjected to 0.15% and 0.3% bile salts, respectively. *L. crispatus* 16/c2, *Ligilactobacillus* *salivarius* (16/c6, 16/i4, A30/i26, 14/i8), *Lactobacillus sp.* 16/i10 and *Limosilactobacillus reuteri* (1/c24 and 12/c8).

The eight chosen lactobacilli were further evaluated for their survival capacity in a medium simulating the GIT conditions of chickens (Figure 4). All strains were able to tolerate acidity and 0.1% (*w/v*) bile salts. However, at 0.3% bile salts, the survival rate was reduced for *Ligilactobacillus* *salivarius* 16/i4 and A33/i26 to 0% and 37%, respectively.

### 2.6. Adhesion Assays

**Figure 5:**
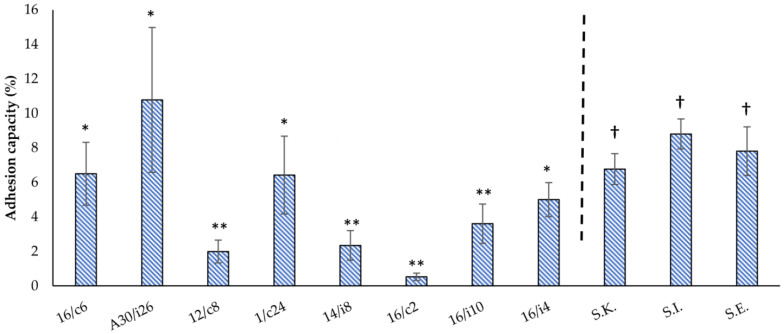
Adhesion capacities of the eight-native poultry-derived lactobacilli strains and the three *Salmonella* serotypes (*S*. Kentucky ST 198 (*S*.K.), *S*. Infantis (*S*.I.) and *S*. Enteritidis (*S*.E.)) to Caco-2 monolayers. The means and standard deviations of the two independent experiments are shown, each with three replicates. The differences between the levels of strain adhesion were evaluated separately for the lactobacilli strains and *Salmonella* serotypes. *Ligilactobacillus* *salivarius* 16/c6, 16/i4 and A30/i26 and *Limosilactobacillus reuteri* 1/c24 revealed no significant differences (*) in their adhesion capacity, a finding which was dissimilar from the four remaining tested strains (**). The differences in the adhesion capacities of *S*. Enteritidis, *S*. Infantis and *S*. Kentucky ST198 were also not significant among the three serotypes (†).

The attachment of the lactobacilli isolates varied from 0.53 to 10.78% (Figure 5). *Ligilactobacillus* *salivarius* (A30/i26, 16/c6 and 16/i4) and *Limosilactobacillus reuteri* 1/c24 showed the highest adhesion abilities (*p* < 0.05) of 10.78% ± 4.2, 6.5% ± 1.82, 5% ± 0.99 and 6.43% ± 2.26, respectively. The remaining *Lactobacillus* spp. 16/i10, *Ligilactobacillus* *salivarius* 14/i8, *Limosilactobacillus reuteri* 12/c8 and *L. crispatus* 16/c2 strains showed no significant differences, with low adhesion capacities of 3.61% ± 1.14, 2.35% ± 0.86, 1.99% ± 0.66 and 0.53% ± 0.21, respectively.

*S*. Infantis, *S*. Enteritidis and *S*. Kentucky ST198 attached to the Caco-2 cells at a percentage of 8.81% ± 0.87, 7.81% ± 1.41 and 6.77% ± 0.89, respectively. No significant difference was found between the different serotypes (Figure 5).

### 2.7. Competition/Exclusion Assays 

**Figure 6:**
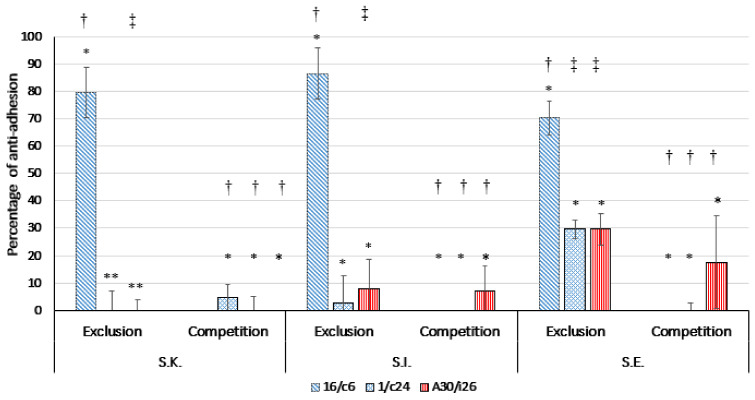
Inhibition of the adherence of *S*. Kentucky ST 198 (S.K.), *S*. Infantis (S.I.) and *S*. Enteritidis (*S*.E.) to Caco-2 cells by *Ligilactobacillus salivarius* 16/c6 and A30/i26 and *Limosilactobacillus reuteri* 1/c24 in competition and exclusion assays. The means and standard deviations of three independent experiments are shown, each with three replicates. (*) *Lactobacillus* strains were fixed and the differences in inhibition were calculated between the three serotypes in the same assay. (*) *p* > 0.05, (**) *p* ≤ 0.05. (†) *Salmonella* serotypes were fixed, and the differences in inhibition were calculated between the three lactobacilli strains in the same assay. (†) *p* > 0.05, (‡) *p* ≤ 0.05.

Three lactobacilli strains that showed the highest adhesion capacity, namely, *Ligilactobacillus* *salivarius* A30/i26 and 16/c6 and *Limosilactobacillus reuteri* 1/c24, were assessed for their ability to compete with the pathogen for the adhesion site on the Caco-2 monolayers (Figure 6). The results showed that none of these strains displayed an effect on the pathogen adhesion to the Caco-2 cells. In the exclusion assays, *Ligilactobacillus* *salivarius* 16/c6 excluded the pathogens to a better degree than *Ligilactobacillus* *salivarius* A30/i26 and *Limosilactobacillus reuteri* 1/c24. The percentages of anti-adhesion to the Caco-2 cells of *S*. Enteritidis, *S*. Infantis and *S*. Kentucky ST198 were 70.30% ± 6.22, 86.57% ± 9.22 and 79.54% ± 9.26, respectively (*p* < 0.05).

### 2.8. Co-Culture Growth Kinetics

**Figure 7:**
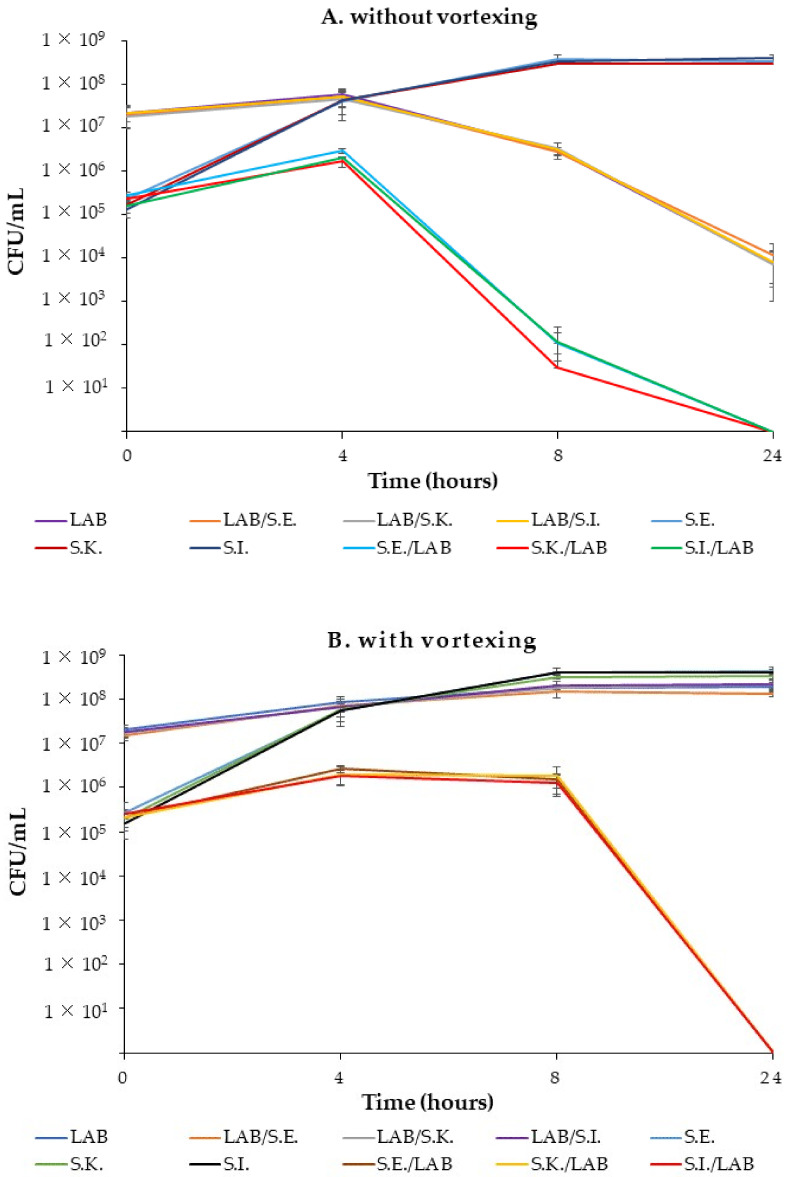
Kinetic growths of the pure cultures and co-cultures of *Ligilactobacillus* *salivarius* 16/c6 and *S*. Enteritidis, *S*. Infantis and *S*. Kentucky ST198 without (**A**) or with (**B**) vortexing. The means and standard deviations of the three independent experiments are shown, each with three replicates.

Since *Ligilactobacillus* *salivarius* 16/c6 was able to inhibit the adhesion of *Salmonella* to the Caco-2 monolayers, its ability to inhibit the growth of *Salmonella* serotypes was assessed in a broth co-culture assay. Pure cultures of the lactobacilli and *Salmonella* serotypes (S.E., S.K., and S.I.) grew very well in the chosen Laptg medium (Figure 7).

In both experiments, without (Figure 7 A) or with (Figure 7 B) vortexing, differences in the CFUs between the control cultures of *Salmonella* (*S*.E., *S*.K., and *S*.I.) and co-cultures (*S*.E./LAB, *S*.K./LAB and *S*.I./LAB) were observed from the early incubation hours. However, the numbers of CFUs estimated from the co-cultures without vortexing were lower than those determined from the vortexed co-cultures and those of the control cultures at 8 h. In fact, the *Salmonella* in the co-cultures increased from 10^5^ to 10^6^ CFU /mL in the first 4 h and then sharply decreased to 10^2^ and 10^1^ CFU/mL, until a negligible cell viability was obtained between 8 h and 24 h. Simultaneously, the *Ligilactobacillus* *salivarius* count decreased from 10^7^ to 10^6^ at 8 h, then was reduced to almost 10^4^ at 24 h in the monoculture (16/c6) and co-cultures (LAB/*S*.E., LAB/*S*.K and LAB/*S*.I.).

In the second set of experiments, *Salmonella* counts from the co-cultures (*S*.E./LAB, *S*.K./LAB and *S*.I./LAB) slightly increased in 4 h and remained constant until 8 h, then decreased to an undetectable level (˂10 CFU/mL) at 24 h. However, the pure cultures of *Salmonella* slightly increased at 8 h and remained constant until the end of the experiments. The lactobacilli counts in the LAB–*Salmonella* mix (LAB/*S*.E., LAB/*S*.K and LAB/*S*.I.) were similar to those of the control monoculture. The pH value in both the mono- and co-cultures dropped from approximately 6.97 to 3.9 at 24 h.

## 3. Discussion 

LAB are considered the principal residents of the GIT, where they provide the host with protection against enteric pathogen colonization (competition for nutrients and secretion of inhibitory substances). These LAB were the focus of many works, substituting the use of probiotics as growth promotors and/or subtherapeutic additives in animal feeds [22]. Numerous factors affect the microbial biodiversity of the poultry GIT, such as the GIT section (ileum or caeca) and the breed, diet and age of the chicken. The microbiota change significantly in the first 2–3 weeks until their stabilization at 5–6 weeks of age. It was found that, when broilers were fed with antibiotic and an additive-free corn-soy diet, 70% of their ileum population was comprised of *Lactobacillus* spp. The use of antibiotics in broilers was shown to induce changes in the composition of the intestinal bacterial community, namely *Ligilactobacillus* *salivarius* [23]. In this regard, our experiments did not detect an important species diversity among the lactobacilli isolates identified. Strains of *Lactobacillus acidophilus*, *Ligilactobacillus* *salivarius* and *Limosilactobacillus fermentum* were permanently found in all birds aged from two days old to market age. Babot and colleagues showed that the most common *Lactobacillus* species were *L. crispatus*, *Limosilactobacillus reuteri* and *Ligilactobacillus* *salivarius*, which was also the case in our findings [24].

In vitro tests have been used to assess the probiotic potential of lactobacilli. The production of hydrogen peroxide, organic acids and bacteriocins are the main strategies of *Lactobacillus* in inhibiting *Salmonella* growth [18]. However, in the present study, hydrogen peroxide production was unlikely to be the cause of this inhibition in the agar diffusion test due to the anaerobic growth conditions of the lactobacilli [25]. The well-diffusion antagonism method did not show any inhibition, thereby excluding the hypothesis of secreted bacteriocins or bacteriocin-like as *Salmonella* inhibitors. Decreasing the pH by organic acid production was likely to be the cause of such an effect [26]. Although the bacteriocin or bacteriocin-like activity produced by LAB is commonly more effective against Gram-positive bacteria, such as *Listeria monocytogenes* [27], the inhibition of the Gram-negative *Salmonella* has also been reported [28].

The adhesion behavior of bacteria is a complex multistep process which includes specific and non-specific ligand–receptor mechanisms [29]. The latter are controlled by physicochemical reactions of the cell wall, including electrostatic and Van der Waals interactions, as well as hydrophobic properties. These are the most reliable long-range non-covalent interactions (Lewis acid–base) due to the surface proteins and lipoteichoic acids covering the peptidoglycan, and conferring a net negative bacterial surface charge in physiological conditions [24]. According to the authors, this feature is strain-specific and may vary depending on the medium, age and surface structures of bacteria. Indeed, considerable variability in the hydrophobicity capacity has been observed in our study, with 65% of the isolates showing high hydrophobicity (70%).

Auto-aggregation properties, together with the co-aggregation ability, of a probiotic strain are necessary for adhering to the intestinal tract by forming a defensive barrier against the colonization of foodborne pathogens [30]. Moreover, the LAB co-aggregating ability might regulate the pathogen microenvironment and stimulate the excretion of antimicrobial substances [31]. *Lactobacillus* spp. also favors many aggregation-promoting factors (APFs) involved in auto-aggregation and/or adhesion in a strain-specific manner [32]. Furthermore, exopolysaccharides (EPS) are believed to play an essential role in cell aggregation, biofilm formation and adhesion. Polak-Berecka and colleagues concluded that the adherence and/or co-aggregation ability of *Lactobacillus rhamnosus* are strongly related to specific interactions based on surface proteins and specific fatty acids, whereas polysaccharides (hydrophilic nature) hinder the adhesion and aggregation by masking protein receptors [33].

Aggregation values have been shown to increase over time, typically at 20 h of incubation, in a strain-dependent manner [34], which is in accordance with our results. All isolates with the (Agg+) phenotype were identified as *Ligilactobacillus* *salivarius*, thus corroborating the findings of Ait Seddik and colleagues, who demonstrated the high auto-aggregation ability of this strain [35]. According to Solieri and colleagues, co-aggregation values below 20% are indicative of a weak co-aggregation ability [36]. Our isolates differed in their co-aggregation abilities (0 to 94.6%), highlighting once again these strain-specific characteristics. 

Another probiotic protective mechanism involves the competition for adhesion sites [37]. *Ligilactobacillus* *salivarius* (16/c6, 16/i4, 14/i8 and A30/i26), *Limosilactobacillus reuteri* (1/c24), *L. crispatus* (16/c2) and *Limosilactobacillus fermentum* (12/c8 and 16/i10) were selected according to their cell hydrophobicity and auto/co-aggregation abilities. The adherence capacity differed significantly between the lactobacilli strains isolated, which is consistent with other studies, showing that this ability is species and strain-dependent [38]. The highest adhesion ability was shown in four isolates of lactobacilli: *Ligilactobacillus* *salivarius* A30/i26 and 16/i4, being highly auto-aggregative and hydrophobic, as well as in *Ligilactobacillus* *salivarius* 16/c6 and *Limosilactobacillus reuteri* 1/c24, showing great co-aggregation and hydrophobicity abilities. *L. crispatus* 16/c2, *Limosilactobacillus reuteri* 12/c8 and *Ligilactobacillus* *salivarius* 14/i8 had the lowest adhesion percentages, despite their high co-aggregation capacities. Interestingly, *Limosilactobacillus* sp.16/i10, a high hydrophobic strain, also exhibited a low adhesion percentage.

The studied parameters, i.e., hydrophobicity, aggregation / co-aggregation and adhesion, illustrated no interrelation. However, some studies mentioned that the cell surface hydrophobicity is related to the attachment to epithelial cells [39,40], while others have excluded this relationship in their analyses [27]. García-Cayuela and colleagues revealed a correlation between auto-aggregation and co-aggregation [29], which disagrees with our results. Del Re and colleagues proposed that auto-aggregation and hydrophobicity are independent characteristics, but both are necessary for adhesion [41]. Multitude interrelated surface factors (fatty acids, surface proteins, LPS and EPS) may have unpredictable effects on adherence, co-aggregation and cell-to-cell interactions [38]. 

Survival in the GIT is a critical probiotic property. Bile tolerance is strain-specific and related to the hydrolase activity [42]. By mimicking the GIT conditions, all the eight lactobacilli strains were capable of growing at 0.1% (*w/v*) bile salts, but two *Ligilactobacillus* *salivarius* strains, namely, A30/i26 and 16/i4, were affected by 0.3%. This concentration is considered critical for screening for resistant probiotic strains [27]. Genes involved in bile salt hydrolysis, *bsh-1* and *bsh-2*, were found to be responsible for the acid and bile tolerance in *Ligilactobacillus* *salivarius* UCC118 [26]. In favor of our findings, a significantly decreasing cell count in most of the *Ligilactobacillus* *salivarius* isolates has been observed when the strains were incubated with a high concentration of bile salts (0.5%), whereas most of the *Limosilactobacillus reuteri* isolates showed a high tolerance [43]. 

*Ligilactobacillussalivarius* A30/i26 and 16/c6 and *Limosilactobacillus reuteri* 1/c24 were selected for their high adhesion abilities and were further evaluated for their potential to compete with the three *Salmonella* serotypes in, or exclude them from, epithelial adhesion using the Caco2 cells as an experimental model. The inhibition of the pathogen adhesion by the three probiotic strains indicated a high variability in a strain-dependent manner. *Ligilactobacillus* *salivarius* 16/c6 significantly inhibited the adhesion of the three *Salmonella* serotypes to the Caco-2 cell monolayers only by exclusion assays, which is in accordance with findings of Campana, Van Hemert and Baffone [38]. The authors suggested that *Ligilactobacillus* *salivarius* W24 could significantly inhibit the adhesion of pathogens to Caco-2 cells only by exclusion. Jankowska and colleagues showed that *L. paracasei* reduced *Salmonella*’s adhesion to Caco-2 cells by 4- and 7-fold in competition and exclusion experiments, respectively [44]. However, the inhibition of *Salmonella*’s attachment to Caco-2 cells by exclusion, as well as by competition, has been frequently reported [37,45,46].

The inhibition of the *Salmonella* serotypes by *Ligilactobacillus* *salivarius* 16/c6 was similarly demonstrated by a mixed co-culture assay. When the co-cultures were tested without vortexing, the kinetic growth results of the lactobacilli and the pathogens confirmed what was previously distinguished by the auto-aggregation and co-aggregation assays and emphasized the ability for these features over time. Indeed, both co-cultures and the *Ligilactobacillus* *salivarius* monoculture revealed a clear supernatant after 8 h of incubation. Additionally, it has been demonstrated that the efficient aggregation and proper settling of flocs are essential for the management of effluent in the activated sludge process [47]. In this regard, such a feature of our strain might be promising in regard to the purification and decontamination of wastewater of slaughterhouses, which is mainly polluted by pathogens and organic materials.

When *Ligilactobacillus* *salivarius* 16/c6 and the *Salmonella* serotypes were subjected to the same co-culture assay but with vortexing, the reduction in the *Salmonella* counts in the mixed cultures co-occurred with a decrease in the pH, which is in accordance with findings of other studies [43]. Some bacterial strains have acid-adaptation systems that enable them to survive at pH < 2 [2]. Other non-negligible antimicrobial factors are involved in the *Salmonella* inhibition, such as competition for nutrients [43] and the contact-dependent inhibition (CDI) mechanism [48]. The latter case, where cell-to-cell contact is required, could be explained by the exchange of and interactions between bacteria mediated by conjugation, secretion systems, contact-dependent inhibition, allolysis and nanotubes. In fact, in our study, the low count was observed at 4 h among the *Salmonella* monocultures and mixed co-cultures.

## 4. Materials and Methods

### 4.1. Isolation and Phenotypic Characterization of Lactobacillus spp.

The different lactobacilli were isolated from the digestive tracts (ileum and cecum) of 16 antibiotic-free healthy broiler groups of different ages (four levels), breeds (four species) and diet formulas (four levels), as well as from 10 antibiotic-treated commercial broilers (Table 3). Experiments coded from 1 to 16 corresponded to the antibiotic-free broiler group, the “A”-coded experiment represents the group of antibiotic-treated commercial broilers, and the sample origin was designated as “i” for ileum and “c” for cecum. Samples of the ileum or cecum content of each category were homogenized at a ratio of 1:10 (10 g of ileum or cecum content in 90 mL of buffered peptone water (Scharlau-Chemie, Barcelona, Spain)). The homogenate was diluted 10^7^-fold and 0.1 mL was plated onto de Man, Rogosa and Sharpe (MRS) agar (Sigma-Aldrich, Burlington, MA, USA). The plates were incubated anaerobically for 3 to 4 days at 37 °C. In total, 210 randomly selected strains were first characterized by Gram staining, motility and the detection of catalase activity. Gram-positive, catalase-negative bacilli were presumptively considered as *Lactobacillus* for further identification. Isolates were preserved in MRS broth with 20% glycerol at −70 °C until further use. All strains were revivified by successive streaking on MRS agar prior to performing any assay.

### 4.2. Salmonella Isolates

The antagonistic activity and co-aggregation ability of the lactobacilli strains were tested on three native avian *Salmonella* strains isolated from our previous study [4,49]. *S*. Enteritidis is the most predominant avian pulsotype causing human illness, whereas *S*. Kentucky ST198 and *S.* Infantis were chosen for their MDR pattern and their high prevalence in Lebanese poultry production. Strains were inoculated into 15 mL tryptic soy broth (TSB) (Sigma-Aldrich) and incubated at 37 °C for 18 h for further analyses.

### 4.3. Assessment of the Lactobacilli Antagonism

The anti-*Salmonella* activities of 210 presumptive lactobacilli were preliminarily screened using the simple spot-on-lawn antimicrobial assay and the agar well-diffusion method [25], with minor modifications. In brief, 10 µL of overnight lactobacilli cultures were spotted onto the surfaces of MRS agar plates and then incubated anaerobically for 18 h at 37 °C. In parallel, an overnight culture of each selected *Salmonella* serotype was inoculated at 10^5^ CFU/mL into 7 mL of TSB soft agar (0.7% agar) and then poured onto the agar plates previously cultured with a strain of lactobacilli. After solidification, the plates were incubated for an additional 18 h at 37 °C under anaerobic conditions. The anti-*Salmonella* activity was evaluated by observing the inhibition zones around lactobacilli spots.

The agar well-diffusion assay was performed to identify the inhibitory substances secreted in the culture supernatants. The lactobacilli isolates showing antagonism were grown overnight at 37 °C in 15 mL of MRS broth. The cell-free supernatants (CFSs) were obtained by centrifugation (4000× *g*, 20 min, 4 °C) and filtration using 0.22 μm-pore Hi-MED syringe filters. The pH of the CFSs was then adjusted to 6.5 by 1 N NaOH. The *Salmonella* isolates were added at 10^6^ CFU/mL to 20 mL of TSB supplemented with 0.75% agar-agar (semi-solid) and then poured into an empty Petri dish. After solidification, 6 mm wells were punched and 50 µL of the CFS was added to each well. The plates were left to settle at 8 °C for 24 h to enable the diffusion of the secreted antimicrobial substances, then incubated at 37 °C for 24 h. The absence or presence of any inhibitory zones was recorded after 24 h of incubation at 37 °C. The two assays were performed in triplicate.

### 4.4. Selection of Strains Based on Their Phenotypic Aggregation

A preliminary visual aggregation screening was performed according to Del Re et al. [41], with minor modifications. In brief, the lactobacilli cultured in the MRS broth were classified into three categories: category I for strains with an aggregation phenotype (Agg+) showing visible aggregates even after vigorous vortexing, category II for strains with constant turbidity and without precipitate (Agg−), and category III for strains with a mixed phenotype forming a precipitate and a clear or small turbid supernatant (Agg+/Agg−).

### 4.5. Species Identification and Phylogenetic Relationships

The 48 selected lactobacilli isolates were identified by 16S rRNA gene sequence analysis. The DNA extraction was achieved with a Qiamp DNA Mini Kit (Qiagen, Hilden, Germany). The amplification of the 16S rRNA gene sequence was performed in a Veriti thermal cycler (Applied Biosystem, CA, USA) under the following cycling conditions:: denaturation at 95 °C for 15 min, 30 cycles of denaturation at 94 °C for 1 min, annealing at 52 °C for 1 min and extension at 72 °C for 2 min, followed by another extension at 72 °C for 7 min. Reaction mixtures (50 μL) were prepared as follows: reaction buffer 10 × (5 μL), 10 mM dNTPs mix (1 μL), 0.5 mM of primer (27F (5′-GTGCTGCAGAGAGTTTGATCCTGGCTCAG-3′) and 1492R (5′-CACGGATCCTACGGGTACCTTGTTACGACTT-3′), bacterial DNA (5 μL) and 2.5 U of HotStarTaq DNA polymerase (Qiagen, Hilden, Germany). Th amplicon separation was completed by electrophoresis at 100 V on 1% (*w/v*) agarose, stained with ethidium bromide in 1 × TBE buffer and purified using the GenElute TM PCR Clean-Up Kit (Sigma-Aldrich) according to the manufacturer’s instructions. The DNA sequencing was carried out on a SeqStudio Genetic Analyzer (Applied Biosystem, USA). The editing was performed with Bioedit (version 7.2.5, 2013), and the 16S rDNA sequences were compared with other sequences using NCBI BLAST (http://blast.ncbi.nlm.nih.gov/Blast.cgi, accessed on 25 April 2022). A phylogenetic tree was assembled using the neighboring methods [50], with the tree builder function of MEGA X [51].

### 4.6. Cell Surface Properties

#### 4.6.1. Auto-Aggregation and Co-Aggregation Assays

Auto-aggregation and co-aggregation capacities of the selected lactobacilli strains, according to their auto-aggregation visual features, were further assessed by spectrophotometric analysis at 4 h and 24 h, as described by Collado and colleagues [34], with minor modifications. An overnight lactobacilli culture (10^8^ CFU/mL) was centrifuged (4000× g, 20 min, 4 °C) and the pellet was washed with phosphate-buffered saline (PBS) at pH 7.1 and then resuspended in the same buffer. The cell suspension (4 mL) was placed into glass bijou bottles and incubated at room temperature for 24 h. The absorbances at 600 nm (A_600 nm_) were measured at different times of incubation (t0, t4 and t24). 

The auto-aggregation percentage was calculated using the following formula: 1 − (A_t_/A_0_) × 100, where A_t_ represents the absorbance at different times (4 and 24 h) and A_0_ the absorbance at time = 0 (t_0_). The aggregation ability was classified according to Del Re and colleagues [41], with minor modifications. Results with values ≥ 65% and ≤10% were considered as highly auto-aggregative and non-auto-aggregative, respectively.

For the co-aggregation assay, mixed cultures with equal volumes (2 mL) of lactobacilli and *Salmonella* strains, as well as monocultures (4 mL), were prepared and incubated at room temperature without agitation. A_600 nm_ were measured at 24 h of incubation.

The percentage of co-aggregation was calculated according to Handley and colleagues [39], as follows: (1− A_mix_/ (A_Sal_ + A_Lac_)/2) × 100, where A_Sal_ and A_Lac_ represent the absorbances of the monocultures, *Salmonella* and lactobacilli, respectively, while A_mix_ represents the absorbance of the mixed culture at 24 h. Values below 20% are indicative of a weak co-aggregation capability [36].

#### 4.6.2. Hydrophobicity Assays

The microbial adhesion to the hydrocarbons (MATH) test was evaluated as defined by Rosenberg and colleagues [52], with slight changes. Lactobacilli cultures were centrifuged, and the pellet was washed with PBS buffer pH = 7.1 and suspended in the same buffer to adjust the concentration to 10^8^ CFU/mL. An equal volume of 2 mL cell culture and xylene (nonpolar solvent) were mixed and vigorously vortexed for 5 min before measuring the A_600 nm_ (A_0_). After incubation at room temperature for 1 h, the aqueous phase was cautiously removed, and its A_600 nm_ (A_1_) was measured. 

The cell surface hydrophobicity (H) was calculated as follows: H% = (1 − A_1_/A_0_) × 100. Isolates with (H) values > 70%, between 50–70% and < 50% were classified as highly, moderately and low-hydrophobic, respectively [53].

### 4.7. In Vitro Cell Tolerance to Gastrointestinal Conditions

The tolerance to gastrointestinal conditions of the eight lactobacilli strains, which were chosen according to their hydrophobicity and auto/ co-aggregation capacities, was assessed according to Babot and colleagues [24], with minor modifications. Overnight lactobacilli cultures were centrifuged (4000× *g*, 4 °C, 20 min) and adjusted to approximately 10^8^ CFU/mL in PBS buffer. A volume of 1.75 mL was inoculated in 2.25 mL of a simulated gastric juice (125 mM NaCl, 7 mM KCl, 45 mM NaHCO_3_, 3 g/L pepsin pH 2.0). After incubation at 41.5 °C (poultry corporal temperature) for 1 h (mean retention time in the proventriculus and gizzard), the suspension was centrifuged and washed twice with PBS buffer. The pellet was then suspended in 3 mL of simulated intestinal juice (NaCl 22 mM, KCl 3.2 mM, NaHCO_3_ 7.6 mM, pancreatin 0.1% *w/v*, bile salts 0.15% or 0.3% *w/v*, pH = 8.00) and incubated at 41.5 °C for 2 h (mean retention time in the small intestine). The concentrations of bile salts were selected to simulate the 0.1 to 1% bile concentration range of the poultry GIT, with approximately 0.25% in the ileum and 0.1% in the cecum [12]. After the serial dilutions, 0.1 mL of the suspensions was plated onto the MRS agar and incubated anaerobically for three days at 37 °C. 

Tolerance to the GIT conditions was evaluated as follows: % survival = (Log_10_ N_1_/Log_10_ N_0_) × 100, where Log_10_ N_0_ is the number of bacterial cells in the PBS, and Log_10_ N_1_ represents the number of viable cells after exposure to gastrointestinal conditions.

### 4.8. Cell Culture

#### 4.8.1. Cell Line and Growth Conditions

The human colorectal adenocarcinoma Caco-2 cell line was used to perform the adhesion assays. Cells were grown in a 75 cm^2^ flask containing Dulbecco’s modified Eagle’s medium (DMEM) (1× DMEM, 1M-1Glutamax, Gibco), supplemented with 10% (*v/v*) heat-inactivated fetal bovine serum (FBS) (Eurobio), 1× non-essential amino acids (NEAA), 100 U/mL penicillin and 10 mg/mL streptomycin (Sigma-Aldrich). Cells were incubated at 37 °C in a humidified atmosphere containing 5% CO_2_ until 80% confluence was reached. Prior to the adhesion assay, 5 × 10^4^ cells were seeded in 24-well tissue culture plates and incubated in the same conditions as mentioned above for 16 days (full differentiation). At the end of the incubation time, the cell line monolayers were washed twice with Dubelcco’s PBS (Eurobio) to remove antibiotics before adding the bacterial suspension.

#### 4.8.2. Adhesion to Caco-2 Cells

Overnight cultures of the selected lactobacilli strains (16/c6, 16/c2, 16/i10, 16/c4, 14/i8, 12/c8, 1/c24 and A30/i26) and *Salmonella* serotypes were centrifuged, washed twice in Dulbecco’s PBS (Eurobio) and suspended in an antibiotic-free DMEM medium at a concentration of 10^8^ CFU/mL. Then, 1 mL of bacterial culture was added to each cell well and incubated for 1 h at 37 °C in a humidified atmosphere containing 5% CO_2_. After that, the supernatant was removed and the wells were gently washed three times with Dulbecco’s PBS buffer to eliminate non-adherent bacteria. Finally, the Caco-2 monolayers were trypsinized with 0.25% trypsin-EDTA solution (Eurobio), and the adherent bacteria were enumerated by plating serial dilutions onto MRS agar medium for *Lactobacillus* and TSA agar medium for *Salmonella*. The adhesion ability was calculated as (N_1_/N_0_) × 100, where N_1_ and N_0_ represent the CFUs of the total adhered and added bacteria, respectively. Two independent experiments were conducted in triplicate for each condition.

#### 4.8.3. Inhibition of the Adhesion of *Salmonella* to Caco-2 Cells

Two different protocols were followed to evaluate the ability of the selected lactobacilli strains to inhibit *Salmonella*’s adhesion to the Caco-2 cells. The *Ligilactobacillus* *salivarius* (16/c6 and A30/i26) and *Limosilactobacillus reuteri* (1/c24) strains were selected based on their adhesion properties. The competition adhesion assay was performed by seeding Caco-2 cell monolayers with a mixed culture of each of the selected lactobacilli (10^8^ CFU/mL) with each of the *Salmonella* strains (10^7^ CFU/mL) in complete DMEM. The *Salmonella* monocultures were used as controls. After an incubation period of 2 h at 37 °C in a humidified atmosphere containing 5% CO_2_, the supernatants with the non-adherent bacteria were removed, and then the Caco-2 cells were trypsinized. The adherent bacterial cells were serially diluted and plated onto TSA agar medium and MRS agar medium to enumerate the *Salmonella* and *Lactobacillus,* respectively.

The ability of the *Salmonella* strains to adhere to the Caco-2 cells in the absence (N_Sal_) and the presence (N_Mix_) of lactobacilli was calculated as follows: 

Anti-adhesion ability% = 1 − (N_Mix_/N_Sal_)% [54].

For the exclusion assays, the Caco-2 cell monolayers were pre-exposed to lactobacilli strains (10^8^ CFU/mL) for 1 h [37]. Then, the Caco-2 cell monolayers were gently washed three times with Dulbecco’s PBS prior to the addition of the *Salmonella* strains (10^7^ CFU/mL) and incubation for 2 h. At the end of incubation time, supernatants with the non-adherent bacteria were removed, and the Caco-2 cells were then trypsinized. The adherent bacterial cells were serially diluted and plated onto TSA and MRS agar media to enumerate the *Salmonella* and *Lactobacillus*, respectively. Two independent experiments for each strain were conducted in triplicate for each condition.

### 4.9. Co-Culture Growth Kinetic Study 

Two series of experiments were carried out to evaluate the effects of *Ligilactobacillus* *salivarius* 16 / c6 on the growth of the *Salmonella* strains in a co-culture model. In the first co-culture experiment, an 18 h-old culture of the 16 /c6 strain (10^7^ CFU/mL) was co-inoculated with each culture of the three *Salmonella* strains (approximately 10^5^ CFU/mL) into 100 mL of Laptg medium (peptone 15 g/L, tryptone 10 g/L, yeast extract 10 g/L, glucose 10 g/L, tween 80 0.1%; all media/chemicals were purchased from Sigma-Aldrich) at pH 6.9 and then incubated in a shaker incubator at 100 rpm at 37 °C for 24 h. Pure cultures of each of the strains served as controls. Before enumeration, the culture was left for 10 min without vortexing to evaluate the auto/co-aggregation capacity of *Ligilactobacillus* *salivarius*. Then, 0.1 mL of supernatant was plated out at different times (0, 4, 8 and 24 h) in triplicate onto the selective media (XLD agar for *Salmonella* and MRS agar for *Lactobacillus*) for counting. The pH of the culture medium was checked regularly. In the second experiment, the bacterial cultures were prepared as described above. Before enumeration, the culture was vigorously vortexed.

### 4.10. Statistical Analyses

Statistical analyses were performed using XLSTAT version 2021.4 (Addinsoft Inc. Paris, France). The surface properties of the forty-eight lactobacilli (*n* = 3) strains were assessed by principal component analysis (PCA). The index of Pearson was used to evaluate the correlation between the six assays, hydrophobicity, auto-aggregation and co-aggregation between a given lactobacilli strain and each of the *S*. Enteritidis, *S*. Infantis and *S*. Kentucky strains. Differences between the results for the adhesion and inhibition by competitive/exclusion were performed by one-way ANOVA, and *p*-values ≤ 0.05 were considered statistically significant.

## 5. Conclusions

This work was the first national tentative attempt to isolate potential candidates from the lactobacilli of Lebanese poultry to act as probiotics. The *Ligilactobacillus. salivarius* 16/c6 isolate that we highlighted here could be used as a potent probiotic dietary supplement in order to reinforce the intestinal microbiota of newly hatched chickens due to its high viability and long persistence in the poultry intestinal tract, as well as its ability to block the adhesion sites against *Salmonella* spp. The adhesion of lactobacilli strains to epithelial cells should also be investigated using the chicken LMH cell line to evaluate its probiotic potential in poultry. The study of these parameters is a crucial step in disseminating such native probiotic strains. However, further in vivo studies are required in order to ultimately better our understanding of how lactobacilli strains interact with and affect the fitness of *Salmonella* in the GITs of chicken hosts.

## Figures and Tables

**Table 1:** Cell surface properties of the eight selected strains of lactobacilli.

Isolates	Visual Aggregation	Auto-Aggregation 4 h (%)	Auto-Aggregation 24 h (%)	% Co-Aggregation with			Hydrophobicity (%)
				*S.* Enteritidis	*S.* Infantis	*S.* Kentucky ST198	
*L. crispatus* 16/c2	Agg+/Agg−	14.46 ± 2.78	58.67 ± 7.62	89.36	75.06	69.66	84.58 ± 1.92
*Ligilactobacillussalivarius* 16/c6	Agg−	9.89 ± 3.63	95.91 ± 2.58	71.07	69.55	94.55	90.26 ± 3.91
*Ligilactobacillussalivarius* 16/i4	Agg+	76.23 ± 3.38	92.95 ± 10.5	82.49	80.45	79.94	82.25 ± 5.84
*Lactobacillus* sp. 16/i10	Agg+/Agg−	6.16 ± 5.53	79.46 ± 1.18	45.60	34.32	63.51	98.36 ± 0.75
*Ligilactobacillussalivarius* 14/i8	Agg+/Agg−	23.14 ± 5.29	73.47 ± 3.67	62.30	70.35	47.54	81.63 ± 1.2
*Limosilactobacillus reuteri* 12/c8	Agg+/Agg−	33.93 ± 6.44	71.86 ± 1.89	83.47	73.87	80.00	52.66 ± 2.98
*Limosilactobacillus reuteri* 1/c24	Agg+/Agg−	13.76 ± 1.87	91.81 ± 7.78	50.43	62.47	58.93	97.53 ± 0.96
*Ligilactobacillussalivarius* A30/i26	Agg+	76.15 ± 3.93	99.63 ± 0.26	49.54	25.71	60.00	98.84 ± 1.34

Values of auto-aggregation and hydrophobicity are the means of triplicate assays with their standard deviations.

**Table 2:** Correlation of Pearson coefficients between hydrophobicity, auto-aggregation and co-aggregation of the 48 lactobacilli isolates. The index of Pearson was used to evaluate the correlation between the six assays, hydrophobicity, auto-aggregation and co-aggregation between the lactobacilli strains and *S.* Enteritidis, *S.* Infantis and *S.* Kentucky stains.

Variables	Hydrophobicity (%)	Auto-Aggregation 4 h (%)	Auto-Aggregation 24 h (%)	Co-Aggregation with *S*. Infantis (%)	Co-Aggregation with *S*. Enteritidis (%)	Co-Aggregation with S. Kentucky (%)
Hydrophobicity (%)	**1**					
Auto-aggregation 4 h (%)	0.2264	**1**				
Auto-aggregation 24 h (%)	0.2665	0.5302	**1**			
Co-aggregation with *S.* Infantis (%)	0.0595	−0.0537	−0.1878	**1**		
Co-aggregation with *S.* Enteritidis (%)	0.1524	0.0202	−0.1880	0.8782	**1**	
Co-aggregation with *S*. Kentucky (%)	0.1496	−0.0208	−0.2181	0.8439	0.8887	**1**

**Table 3:** List of coded experiments numbered according to age, breed and diet formula of the broilers and hens deprived of any antibiotics and feed additives. The A-coded sample refers to non-antibiotic-free commercial broilers.

Experiment Number	Category, Age	Breed	Diet Formula
**1**	Broiler, 35 days	Cobb	High starch diet: corn 60%, soya 20%, wheat 20%
**2**	Broiler, 35 days	Cobb	High protein diet: soya 40%, corn 40%, wheat 20%
**3**	Broiler, 35 days	Cobb	High gluten diet: wheat 60%, soya 20%, corn 20%
**4**	Broiler, 35 days	Ross	High starch diet: corn 60%, soya 20%, wheat 20%
**5**	Broiler, 35 days	Ross	High protein diet: soya 40%, corn 40%, wheat 20%
**6**	Broiler, 35 days	Ross	High gluten diet: wheat 60%, soya 20%, corn 20%
**7**	Broiler, one day	Cobb	High starch diet: corn 60%, soya 20%, wheat 20%
**8**	Broiler, one day	Cobb	High protein diet: soya 40%, corn 40%, wheat 20%
**9**	Broiler, one day	Cobb	High gluten diet: wheat 60%, soya 20%, corn 20%
**10**	Broiler, one day	Ross	High starch diet: corn 60%, soya 20%, wheat 20%
**11**	Broiler, one day	Ross	High protein diet: soya 40%, corn 40%, wheat 20%
**12**	Broiler, one day	Ross	High gluten diet: wheat 60%, soya 20%, corn 20%
**13**	Layer, 69 weeks	Isa Brown	Normal feed: corn 40%, soya 32%, wheat 20%
**14**	Layer, 69 weeks	Isa White	Normal feed: corn 40%, soya 32%, wheat 20%
**15**	Layer, 27 weeks	Isa Brown	Normal feed: corn 40%, soya 32%, wheat 20%
**16**	Layer, 27 weeks	Isa White	Normal feed: corn 40%, soya 32%, wheat 20%
**A**	Broiler, 35 weeks	Ross	Normal feed: corn 40%, soya 32%, wheat 20%
